# Graphene Quantum Dots from Agricultural Wastes: Green Synthesis and Advanced Applications for Energy Storage

**DOI:** 10.3390/molecules29235666

**Published:** 2024-11-29

**Authors:** Pierfrancesco Atanasio, Rubia Y. S. Zampiva, Luca Buccini, Corrado Di Conzo, Anacleto Proietti, Francesco Mura, Annalisa Aurora, Andrea G. Marrani, Daniele Passeri, Marco Rossi, Mauro Pasquali, Francesca A. Scaramuzzo

**Affiliations:** 1 Department of Basic and Applied Sciences for Engineering (SBAI), Sapienza University of Rome, Via A. Scarpa 14, 00161 Rome, Italy; pierfrancesco.atanasio@uniroma1.it (P.A.); rubia.zampiva@uniroma1.it (R.Y.S.Z.); luca.buccini@uniroma1.it (L.B.); anacleto.proietti@uniroma1.it (A.P.); francesco.mura@uniroma1.it (F.M.); daniele.passeri@uniroma1.it (D.P.); marco.rossi@uniroma1.it (M.R.); mauro.pasquali@uniroma1.it (M.P.); 2 Department of Applied Science and Technology (DISAT), Polytechnic of Turin, Corso Castelfilardo 39, 10129 Torino, Italy; corrado.diconzo@polito.it; 3 Research Centre for Nanotechnology Applied to Engineering, Sapienza University of Rome (CNIS), Piazzale A. Moro 5, 00185 Rome, Italy; 4 Department of Energy Technologies and Renewable Sources C.R. ENEA Casaccia, Via Anguillarese 301, 00123 Rome, Italy; annalisa.aurora@enea.it; 5 Department of Chemistry, Sapienza University of Rome, Piazzale A. Moro 5, 00185 Rome, Italy; andrea.marrani@uniroma1.it

**Keywords:** graphene quantum dots, rice husk, supercapacitors, Li-ion batteries, nanomaterials, energy storage, waste-to-energy

## Abstract

Carbon nanostructures are highly promising materials for applications in a variety of different fields. Besides their interesting performances, the possibility to synthesize them from biowaste makes them an eco-friendly resource widely exploitable within a circular economy context. The present work deals with the green, one-pot synthesis of graphene quantum dots (GQDs) from carbon aerogels (CAs) derived from rice husk (RH). After having obtained CAs upon purification of RH, followed by gelification and carbonization of the resulting cellulose, the one-pot solventless production of GQDs was obtained by ball milling. This method determined the formation of crystalline nanostructures with a diameter of around 20 nm, which were analyzed via scanning electron microscopy, transmission electron microscopy, atomic force microscopy, X-ray diffraction, and Raman spectroscopy to obtain a full morphological and structural characterization. GQDs were used as electrode materials for supercapacitors and Li-ion batteries, showing the ability to both accumulate charges over the surface and intercalate lithium-ions. The reported results are a proof of principle of the possibility of exploiting GQDs as support material for the development of advanced systems for energy storage.

## 1. Introduction

Carbon nanomaterials derived from biowaste are being extensively explored as eco-friendly resources in the pursuit of sustainable technological advancements [1]. Together with industrial waste [2], biowaste often serves as a source to produce graphitic structures, following the principles of circular economy and waste valorization [3]. Among the biowaste sources, rice husk (RH) has garnered significant research interest in both academic and industrial contexts due to its abundant availability at minimal cost and its substantial potential for conversion into high-quality graphene [4,5]. Studies involving RH have shown a significant increase since 2020 and continue to rise, with no indication of a slowdown [5].

Current rice production worldwide is estimated to be 515.83 million metric tons (milled basis) [6,7]. RH is a by-product of the rice milling process, accounting for 20 wt% to 33 wt% of dried rough rice. Between 30 wt% to 50 wt% of the RH composition is organic carbon [8,9]. With the ongoing focus on the environment, the random disposal and open burning of RH have been drastically reduced, and various alternative uses for this biowaste are being developed. RH can be found as a heavy metal adsorbent, an insulator in refractory brick and steel industries, and a filler in pigments, rubber, cement, and concrete [9,10]. It is also utilized in the electronic industries, solar grade, and energy fields [11].

On the energy storage field, the most popular commercially available systems are Li-ion batteries, characterized by robustness and safety. Depending on the application, the cathode can be made of oxides or salts like LiCoO_2_ or LiFePO_4_, while graphite is generally used as material for the anode [12]. However, the rapid development of electronic tools creates a continuous need for increasingly high-performance energy storage devices. Consequently, research on new electrode materials is currently among the hot topics in the field [13,14,15,16]. Moreover, lithium is a critical raw material, and it is consequently necessary to go beyond lithium technology, exploring the possibility of using different systems like lithium–sulfur batteries, Na-ion batteries, and supercapacitors [17,18,19].

Supercapacitors have gained attention in the scientific community due to their high-power density, long cycle life, outstanding cycling stability, and low cost [20]. However, the broad utilization of these devices has been challenging because of their relatively low energy density. Currently, multiple research groups are working on producing high-energy-density supercapacitors by either improving specific capacitance or increasing the voltage window [21,22]. Therefore, the type and nature of electrode materials have a critical impact on the electrochemical properties of supercapacitors [23].

One promising approach to producing electrodes for supercapacitors is the utilization of the organic carbon content present in RH. As indicated in the literature, various graphitic structures can be derived from RH biomass [5,24], including carbon-based materials classified as zero-dimensional (0D), such as graphene quantum dots (GQDs). GQDs are formed by highly crystalline and few-atom-thick graphene with sp2-hybridized carbon and dimensions under 100 nm. The thickness of the GQD structure is close to the single atom [25]. Therefore, unique properties deriving from strong quantum confinement and edge effects of quantum dots are expected. GQDs present photoluminescence, high conductivity, chemical inertness, excellent stability, and low toxicity. These dots contain energy band gaps and delocalized charge carriers within their nanoscale structure. There are also plenty of edge sites for functionalization, resulting in additional properties with a high specific surface area and excellent ionic transporting ability [26,27,28]. Current research on these structures involves both top-down and bottom-up synthesis approaches and the applications can range across a wide set of topics like sensors, solar cells, bio imaging, catalysis, and optoelectronic devices [29,30,31].

Considering the abovementioned GQDs’ properties, it is reasonable to expect them to be highly functional as supercapacitor electrode material with elevated capacitance and stability. Furthermore, as for the supercapacitors, those properties make GQDs interesting for application as electrodes in various electrochemical devices [32,33].

Based on the presented information, this study proposes the utilization of RH as a precursor for synthesizing high-purity GQDs and their application in energy storage. The literature outlines various routes for the production of GQDs from biomass in general [34,35] and specifically from RH [10,36,37]. However, these methods typically involve rigorous chemical treatments and often more than one passage at high temperature. The processing of RH includes a step in which amorphous carbon is obtained through a purification process that removes silicon and other impurities inherent to the natural material, and the resulting carbonaceous material typically undergoes a second step, which frequently involves additional elevated temperatures and chemical treatments to produce GQDs [4].

With a focus on eco-sustainability, a top-down synthesis route based on two simple steps for producing high-purity GQDs by recycling RH is presented in this paper. The first step consists of the formation of a carbon aerogel (CA) after cellulose purification and gelification. The second step involves a mechanical-energy-based process, during which the obtained CA is subjected to ball milling, resulting in the production of GQDs with an approximate size of 20 nm. This approach utilizes a recyclable source and employs a green synthesis passage, ensuring that product quality remains uncompromised. Ball milling further proved to be a non-toxic, pollution-free technique which can be extended to a wide variety of starting matrices to obtain nanostructured materials.

To investigate the potential of the obtained GQDs in electrochemical devices, and given the well-known importance of obtaining a complete morphological and structural characterization to fully exploit the potential of materials for energy applications [38,39], the nanostructures were analyzed by different techniques, e.g., scanning electron microscopy (SEM), atomic force microscopy (AFM), transmission electron microscopy (TEM), X-ray diffraction (XRD), Raman spectroscopy, Fourier transform infrared spectroscopy (FTIR), X-ray photoelectron spectroscopy (XPS), and thermal gravimetric analysis (TGA). Finally, as a proof of principle, the synthesized 0D materials were used as carbonaceous electrodes for supercapacitors and lithium-ion batteries. Despite GQDs being more often coupled with a second active material component, the shown physico-chemical properties and morphology suggest that GQDs produced via the proposed method could demonstrate superior performance compared to those reported in the literature for single-component GQD electrodes. The production of high-quality single-component GQD electrodes enables the optimization of a starting material, which can still subsequently be treated or combined with various solutions to achieve maximum performance.

## 2. Results

### 2.1. GQD Characterization

**Figure 1:**
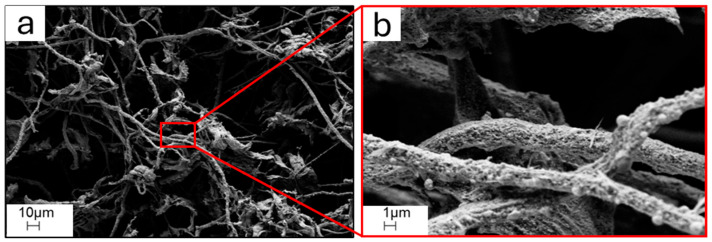
(**a**) SEM micrography 1k× magnification of starting CAs; (**b**) corresponding 10k× magnification of the area selected in red.

The CAs obtained from RH and used as precursors for the synthesis of GQDs were characterized by carbonized cellulose in the shape of interconnected networks of both fibers and thin platelets, whose typical SEM appearance is shown in Figure 1.

**Figure 2:**
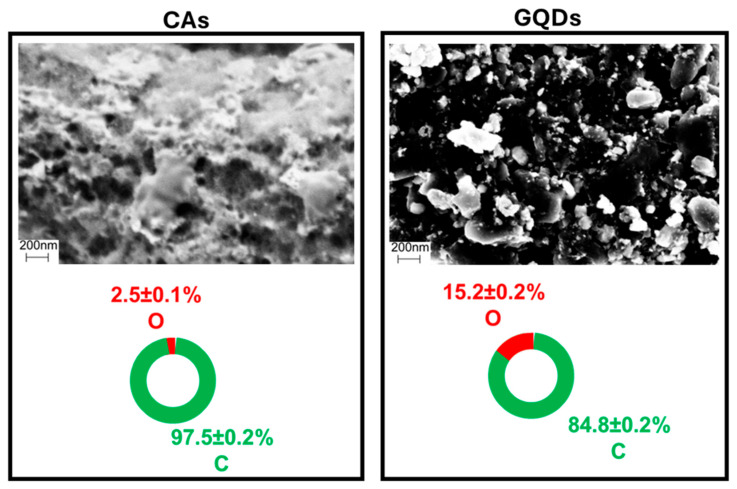
SEM-EDX of starting CAs and GCDs in bulk aggregate powder, pointing out the morphological and compositional differences in terms of carbon and oxygen between precursor and final product.

Upon the facile one-step synthesis of GQDs, i.e., ball milling of CAs, about a 10% increase in sample mass was observed. Since the synthesis step occurs in the air, such an increase is probably due to a reaction with environmental oxygen induced by the high-energy impacts of the ball milling spheres against the carbonaceous substrate. Such a process may be considered responsible for the formation of oxygen-rich groups along the particles’ surfaces. This hypothesis is supported by SEM-EDX data acquired on bulk aggregate powder, which gave information about the qualitative and quantitative elemental composition of GQDs. Upon comparison with the starting CAs, reported in Figure 2, the change in morphology is coupled with an increase in the oxygen percentage, rising from an average of 2.5% up to more than 15%.

**Figure 3:**
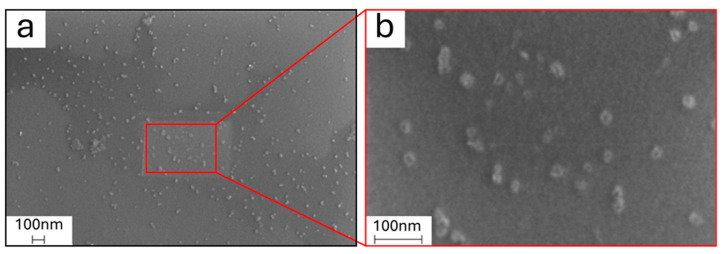
(**a**) SEM micrographs of graphene quantum dots dispersed on a silicon wafer after dilution in water at 100k× magnification and (**b**) 400k× magnification.

Besides as a bulk aggregate powder, GQDs were also observed using SEM-EDX upon dispersion over a silicon wafer substrate after suspension in water, to properly observe single nanoparticles and to accurately determine their diameter distribution. As observable in Figure 3, GQDs appear to have an irregular almost circular shape, while an average diameter of 27 ± 9 nm was estimated for them by ImageJ software.

**Figure 4:**
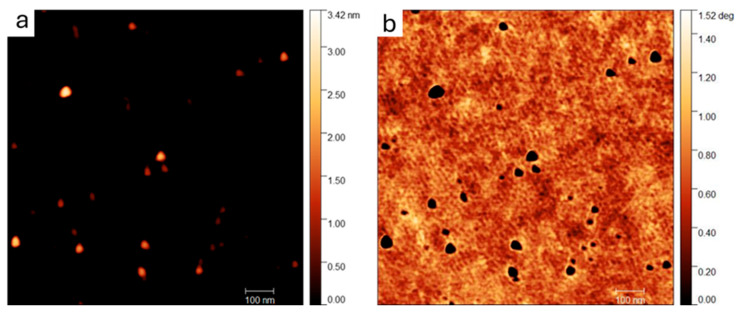
(**a**) Typical topography and (**b**) phase 1 µm × 1 µm AFM images of GQDs deposited on Si wafer from a diluted suspension in water.

The morphology and dimension of graphene quantum dots were also investigated using atomic force microscopy. The typical topography and phase-recorded images of the diluted sample dispersed on a Si wafer are shown in Figure 4. In good agreement with SEM, the AFM of dispersed graphene quantum dots confirms the irregular shape of the nanostructures, with an average diameter estimated using ImageJ to be around 30 ± 6 nm. According to a *t*-test performed on the two sets of data collected with SEM and AFM, the estimated average diameters are statistically identical; however, the slightly higher values obtained by AFM are reasonable, considering the tip-sample convolution due to the interaction during the scan.

**Figure 5:**
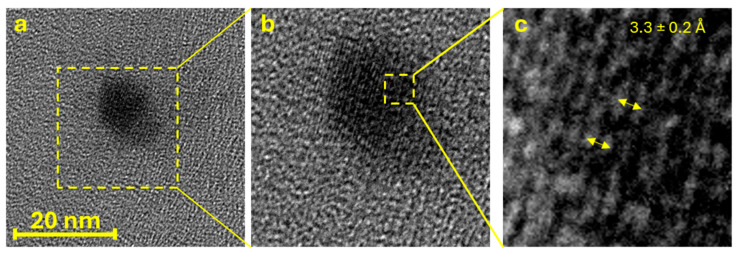
(**a**) HRTEM image of a GQD, (**b**) magnification on its crystalline planes, and (**c**) magnification on the interplanar distance among crystalline planes.

To further validate the identification of the observed sample as GQDs and obtain additional information about their morphology and crystallinity, HRTEM measurements were performed on a highly diluted suspension (1:106). As observable in Figure 5, HRTEM shows nanostructures with irregular circular shapes and visible crystalline planes (insert b). Based on HRTEM data, an average diameter of 22 ± 1 nm was calculated for the dots. This value is slightly lower than the values obtained with SEM and AFM analysis but is still in good agreement with them. This is reasonable, considering the abovementioned tip-sample convolution for AFM and the low sensibility of SEM at high magnification, which causes a blur in the imaging, while HRTEM is certainly a more accurate technique for particularly small carbon-based samples. By observing the crystalline plane distance, an average interplanar distance of 3.3 ± 0.2 Å was estimated, which is in good agreement with values in the literature for graphene plane distances in graphite (3.35 Å) [40,41]. However, despite being visible, the crystalline planes seem to show occasional interruptions and spots (i.e., the alternation of white and black lines that show the sample crystallinity is not totally regular), which could indicate some residual disorder and irregularities in the crystalline structure.

**Figure 6:**
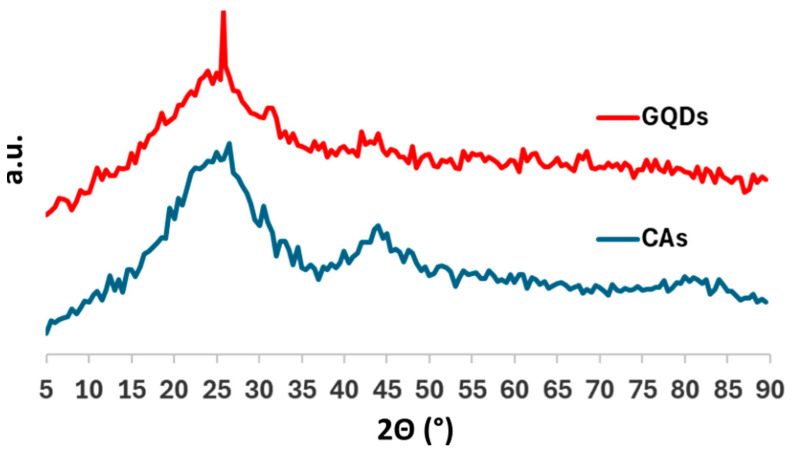
Diffractograms of starting carbon aerogels (red line) and graphene quantum dots (blue line).

Aiming to more precisely evaluate sample crystallinity and amorphous content and collect structural information concerning both long- and short-range order, XRD and Raman spectroscopy were utilized. The obtained results were compared to the starting material (i.e., carbon aerogel) to evaluate modification during synthesis. According to XRD diffractograms shown in Figure 6, while the starting CAs showed no clear long-range order or structure, it is possible to observe a sharp peak at 26.57° in 2Θ, assigned to the (002) signal of graphite due to the repetition and stacking of graphene sheets. Such a clear change indicates that during ball milling, the impact between the powder and spheres not only promotes the formation of external oxygen-rich functional groups, but also induces crystallization of the resulting GQDs; therefore, the final material has a consistently higher crystalline structure compared to CAs. Moreover, by using Bragg’s equation, it is possible to convert the 2Θ value of the peak in the interplanar distance, obtaining the value of 3.35 Å, which is exactly the reported interplanar distance in graphite and further validates the crystallinity and interplanar distance distribution observed by HRTEM. Despite having a higher crystallinity degree than CAs, GQD powder still shows a strong amorphous signal between 18° and 28°, thus indicating the presence of a disordered fraction in samples. Finally, the Scherrer analysis was applied to GQD diffractogram to estimate the average dimension of the crystalline grains according to the equation:

(1) $$L=\frac{K\lambda }{\mathrm{FWHM}\;\cos(\Theta )}$$

where L is the crystalline grain dimension, K is the shape coefficient (0.94 for spherical particles), λ is the wavelength of the copper radiation (1.54 Å), FWHM is the full width at half maximum of the main (002) in radiant, and Θ is the position of the peak. According to this calculation, the average size of the crystallites is around 18.1 nm. It is worth noting that the Scherrer equation has already been successfully used for graphene fragments [42], and our result is comparable to the value already reported in the literature for similar GQDs [43].

**Figure 7:**
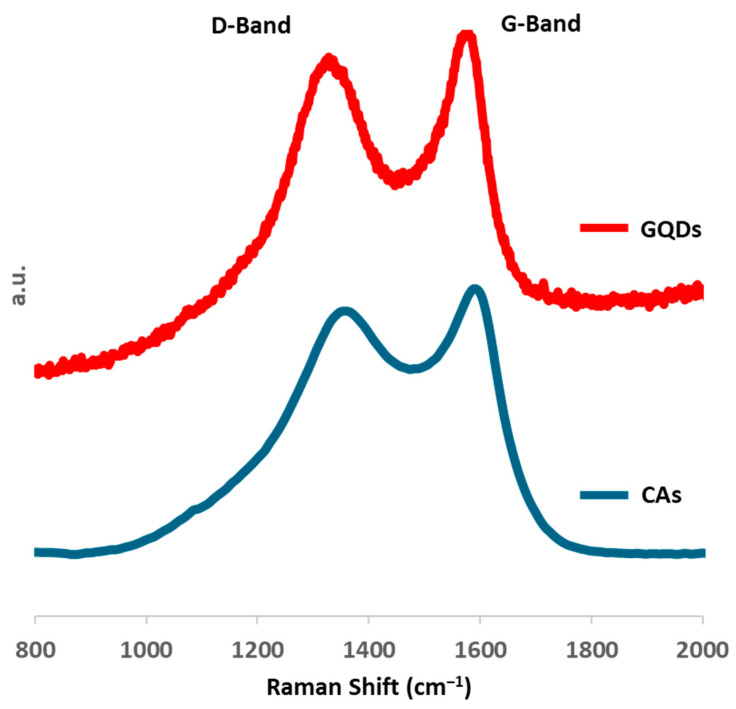
Raman spectra of starting CAs (blue line) and GQDs (red line).

The Raman spectra of starting CAs and obtained GQDs are reported in Figure 7. Both samples show the typical spectra of carbonaceous graphitic materials. However, there are differences in the band widths and both the D- and G-bands of starting CAs appear significantly broader, thus indicating a lower degree of disorder within graphene quantum dots compared to their precursor [44]. The position of the bands has been investigated with a four Gaussian fitting using Breit–Wigner–Fano lines [45]: according to the fitting, D- and G-band positions are, respectively, 1333 and 1587 cm^−1^ in GQDs, and 1307 and 1564 cm^−1^ in CAs (see Supplementary Information, Figures S1 and S2). Furthermore, the ratio between the intensity of the D-band and the G-band (i.e., I_D_/I_G_) decreases from 1.09 for CAs to 1.04 for GQDs. Based on the higher Raman shift in GQDs of G-band and its lower I_D_/I_G_ around 1, the three-stage model [46] suggests a negligible percentage of sp^3^ bonds and a more graphitic structure of GQDs, thus confirming the increase in crystallinity suggested by peak broadness, even though the hysteresis of the amorphization curve could potentially underestimate the percentage of sp^3^ bonds in CAs.

The comparison of XRD and Raman data suggests that both samples shared the same short-range order. The phase transition during the reaction, i.e., the crystallization of GQDs into a more ordered graphite with a long-range order, seems to be still incomplete, leaving defects and irregularities among the graphene layers. The latter experimental evidence might confirm the irregularities and disordered spots shown in the crystalline planes observed in HRTEM imaging.

**Figure 8:**
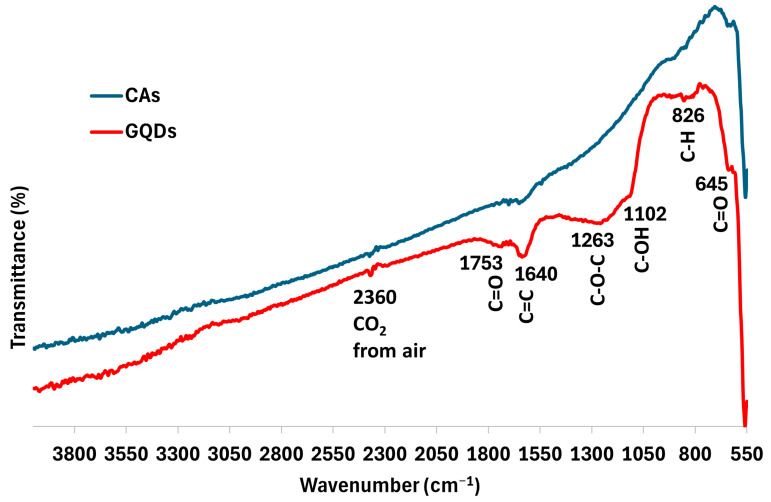
ATR-FTIR spectroscopy of the samples CAs (blue line) and GQDs (red line).

The samples’ surfaces were also examined by FTIR spectroscopy (Figure 8). The spectra demonstrate an evident increase in oxygenated functional groups on the GQDs compared to the CA surface. This result is in accordance with the oxygen increase observed by EDX. Bands attributed to the C=O of carboxylic acid and carbonyl groups are shown at ~1753 and 645 cm^−1^ [47], while the bands at ~1263 and 1102 cm^−1^ belong to the stretching of C–O–C bonds [48] and C–OH vibrations [49], respectively. The band around 826 cm^−1^ is associated with the symmetric and asymmetric vibration modes of C–H groups [50]. C-H and C=O bands are also present in the CA spectrum, but with less intensity when compared to GQDs. Furthermore, a band at ~1640 cm^−1^ is also observed for both samples, with higher intensity in the GQD spectrum. This band can be associated with C=C stretching and indicates the formation of new sp^2^–hybridized (graphitic) carbon bonds [48,49] for the ball-milled sample, in agreement with the results found by XRD and Raman spectroscopy. Both spectra present a similar band at 2360 cm^−1^ caused by CO_2_ from the air [51].

**Figure 9:**
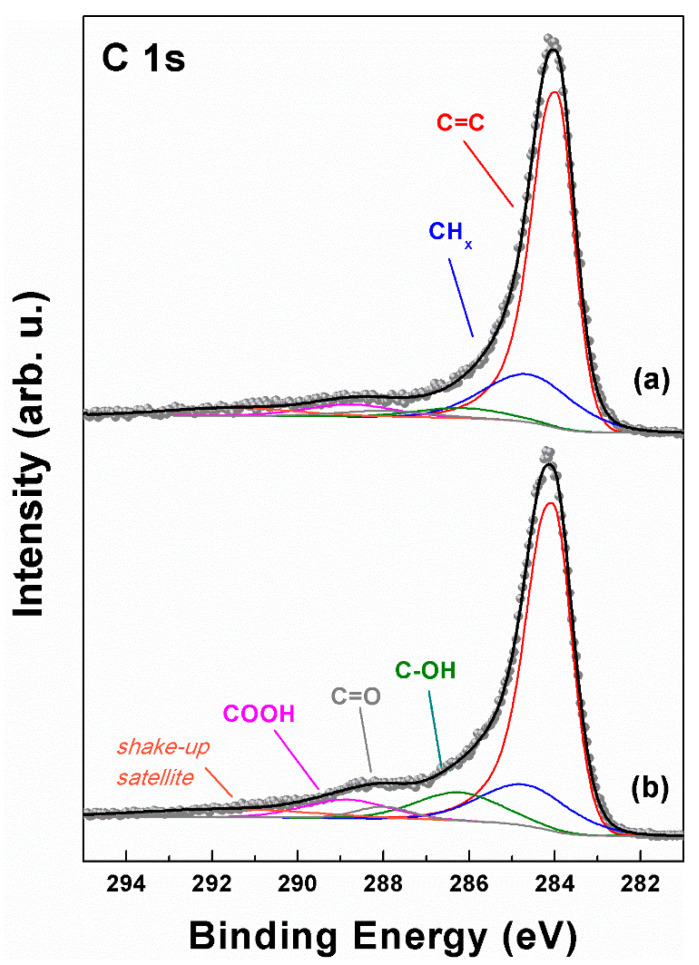
XPS C 1s ionization region of (**a**) CA and (**b**) GQD samples. Raw data are displayed with dots, while the curve fitting reconstruction is represented by continuous colored lines.

XPS measurements were run on both CA and GQD samples in order to ascertain their chemical composition and the possible presence of oxygenated functional groups within the carbon atom network. XPS reported in Figure 9 shows the C 1s ionization region for both samples with the corresponding curve fitting results. In both cases, a common line shape can be identified, with a predominant peak at low binding energy (BE), followed by a series of weak contributions from various oxygenated functional groups. The lowest energy peak is located at 284.0 eV (red curves in Figure 9) and displays a moderate asymmetric tail towards high BEs. According to these features, this contribution can be assigned to C=C bonds within a network of conjugated π bonds, as those typical of graphitic materials [52,53,54,55]. The next contribution at 284.7 eV (blue curves) can be assigned to aliphatic defective sp^3^ C atoms [56], whereas the higher BE peaks are representative of C atoms bound to oxygen, such as C–OH (hydroxyl, 286.2 eV, green curves), C=O (carbonyl, 288.0 eV, gray curves), and COOH (carboxyl, 288.8 eV, magenta curves) [54,55,57,58,59]. An additional broad and weak signal can also be detected around 290 eV, attributable to a π-π* shake-up satellite typical of π-conjugated systems.

Despite having a common sequence of chemical contributions to the spectrum, the two samples displayed slightly different intensities in the oxygenated carbon atoms portion of the spectrum, with the GQD sample being more oxygen-rich than the CA sample, especially in the C-OH and COOH groups. The area of the peaks resulting from the curve fitting of the C 1s region allowed us to extract an atomic O/C ratio (R_O/C_) through Equation (4) [52] (see Experimental), which resulted in 0.16 for CAs and 0.24 for GQDs. The higher amount of oxygenated functional groups in GQDs is in line with the SEM-EDX and FTIR findings, and can be traced back to a probable oxidation of the sample surface during the ball milling process.

**Figure 10:**
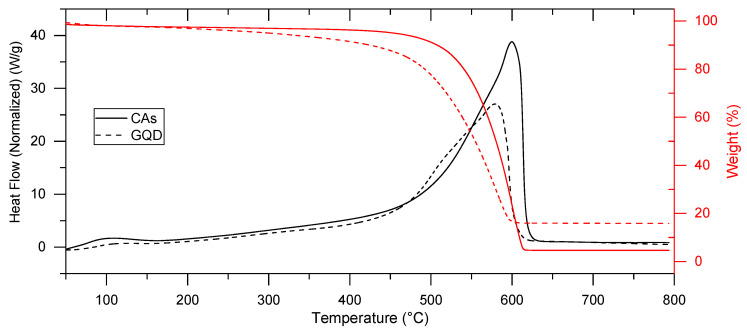
TGA-DTA curves of CAs (solid line) and GQDs (dashed line) carried out in technical air.

TGA (Figure 10) shows that both samples have a slight weight loss due to adsorbed water before 100 °C (about 2.0% for CAs and 8.9% for GQDs) and a single decomposition step over 400 °C with a consistent weight loss. The CA curve shows a decomposition weight loss at the extrapolated onset temperature of 543.1 °C, which corresponds to a DTA exothermic peak with a maximum at 580.2 °C. GQD samples are more thermally unstable, and the weight loss starts slightly from 200 °C if compared with the CA curve. This phenomenon is associated with the increase of labile oxygen-containing functional groups in GQDs during the ball milling process [60]. For GQDs, the decomposition occurs at the onset temperature of 494.6 °C, and the corresponding DTA exothermic peak shows its maximum at 457 °C. The exothermic decomposition peaks for both samples are assigned to the combustion of the carbon skeleton of graphene oxide [60,61].

At 800 °C, the residue is 4.6% of the initial weight for CAs and 16.5% for GQDs. According to the above-presented chemical analysis, only carbon, oxygen, and hydrogen are present in the samples, and in this way, the remaining mass is attributed to graphitic carbon [61]. Those values indicate an increase of almost 28% in ordered graphitic carbon after the ball milling process.

### 2.2. GQD-Based Electrode Materials for Energy Storage

Graphene quantum dots have been studied as active material components in both supercapacitors and in lithium-ion devices in order to investigate the potential of 0D graphitic materials for electrochemical devices.

**Figure 11:**
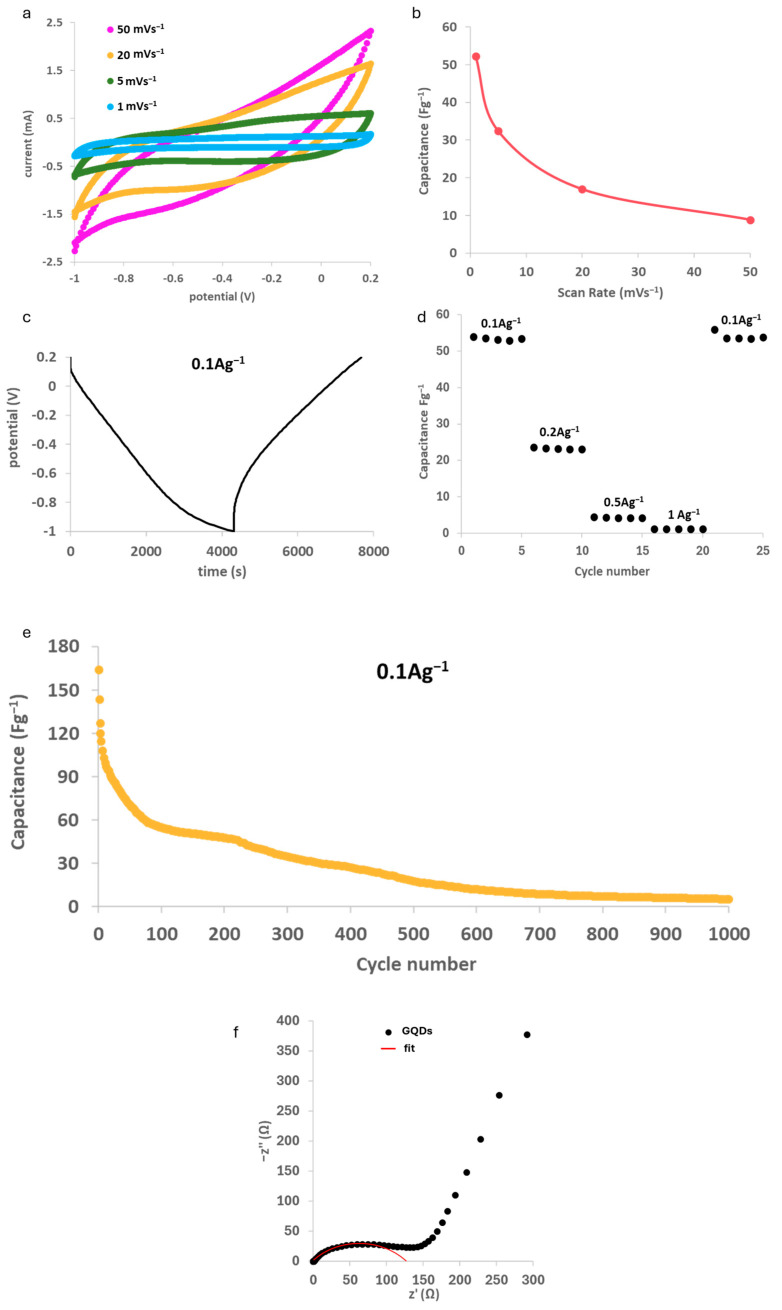
Electrochemical performance of GQDs in supercapacitors: (**a**) cyclic voltammetry at different scan rates; (**b**) capacitance evaluated by CV; (**c**) potential profile in time in galvanostatic cyclations; (**d**) capacitance values calculated at different currents; (**e**) device life; and (**f**) electrochemical impedance spectroscopy showing GQD behavior (black dots) and fitting curve (red line).

Cyclic voltammetry at different scan rates for symmetrical GQD electrode supercapacitors is shown in Figure 11a. At low scan rate values, the curves show a quasi-rectangular shape with no visible peak typical of supercapacitors, which seems almost lost at a high scan rate. In any case, the curves are symmetrical to the horizontal axis, and this can indicate a good reversibility of the charge accumulation. The absence of visible peaks indicates the absence of faradic reactions or side processes due to the functionalization of the surface during GQD synthesis, and the process appears totally capacitive with no transformations occurring in the device. Capacitance has been evaluated for different scan rates by CV using Equation (2):

(2) $$C=\frac{\int IdV}{\varepsilon mV}$$

where C is the specific capacitance, I is the current, V is the potential window, m is the mass of the active material in the electrode, and ε is the scan rate.

Results are shown in Figure 11b, where the range of values goes from 10 Fg^−1^ at 50 mVs^−1^ to around 53 Fg^−1^ at 1 mVs^−1^, with the material decreasing in capacitance when forced to work under more stressful conditions.

Galvanostatic cyclations were also performed: the trend of potential during the first cycle of discharge and charge at 0.1 Ag^−1^ is shown in Figure 11c. A quasi-triangular shape is maintained and confirms the absence of faradic reactions already proved during cyclic voltammetry. However, at the beginning of the charging process, there is a potential jump of almost 0.2 V, after which the potential increase is rather linear. This indicates a consistent irreversible loss of capacitance which could in principle hinder the device’s life after many cycles.

In this case, the capacitance is calculated by using Equation (3):

(3) $$C=\frac{I∆t}{mV}$$

where C is the specific capacitance, I is the constant discharging current density, Δt is the discharging time, m is the mass of active material in the electrode, and ΔV is the potential window in the galvanostatic discharge curves. Capacitance has also been observed at different currents applied and it can be seen in Figure 11d that performance is not hindered by higher currents applied.

The capacitance trend over the charge/discharge galvanostatic cycle, i.e., the supercapacitor life cycle of a device with a GQD-based electrode cycling at 0.1 Ag^−1^ current density, is reported for a thousand cycles in Figure 11e. From this plot, the effect of the abovementioned irreversible capacitance loss is clearly visible: after outstanding values in the first cycle (over 100 Fg^−1^), capacitance fades until it reaches a stable trend in the range between the 50th and the 200th cycles around 55 Fg^−1^. Moreover, a second fast decrease in capacitance occurs, and after 1000 cycles, the sample reaches a performance of around 5.2 Fg^−1^. Although it is clear that our GQDs, as tested under experimental settings, are not suitable for commercial applications, the results obtained in galvanostatic cyclations are definitely comparable, and even superior, to those reported in the literature for single-component GQD electrodes with a similar average particle size [62]. Çolak et al. [62], using a bottom-up synthesized material from citric acid, only obtained a capacitance of 32.08 Fg^−1^ at 0.1 Ag^−1^ with an electrode made using only carbon dots as the active material. Similarly, and both using a top-down synthesis, Dharmalingam et al. [63] only obtained a maximum capacitance of around 30.5 Fg^−1^ at 0.1 Ag^−1^, while Baslak et al. [64], using biomass (*Stachys euadenia*), managed to obtain carbon quantum dots of similar sizes compared to us (18–22 nm), but their performance in symmetric capacitors using KOH 6 M in cyclic voltammetry is significantly lower than our material (around 2 Fg^−1^), with no crystalline order of the particles. A schematic comparison is reported in Table 1.

It is worth noting that higher capacitance values reported in the literature for similar materials refer to systems in which the GQDs are coupled with other active carbonaceous components, e.g., carbon nanotubes or graphene oxide or even metallic compounds, giving rise to functional hybrid materials with enhanced performances. Therefore, the reported preliminary analysis demonstrates that the present system shows ample room for improvement, especially if coupled with other active materials. An example is in the work of Ji-Shi et al. [65], where various morphologies of composite material with NiCo_2_O_4_ were successfully prepared and stable capacitance above 800 Fg^−1^ were achieved even at currents above those examined in this paper.

Moreover, to further investigate the electrochemical behavior of GQDs, EIS analysis was applied and resistances around 0.6 Ω and 128.5 Ω were calculated by the fitting showed in Figure 11f.

GQDs were also tested in lithium-ion batteries to evaluate the efficacy of lithiation inside a 0D material with a high specific surface. GQDs alone typically do not exhibit high performance as anode materials due to the significant contact resistance between GQDs and their mismatch in volume concerning lithium intercalation [66].

**Figure 12:**
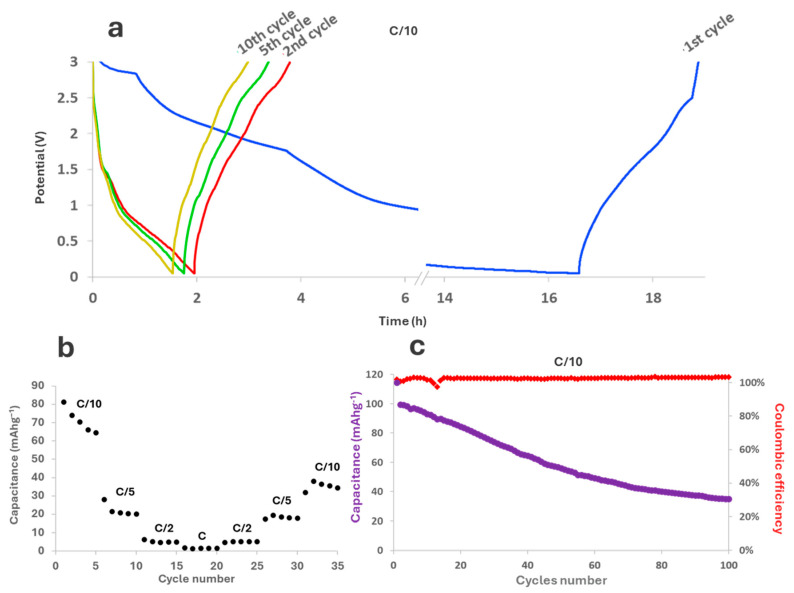
Electrochemical performance of GQDs as lithium-ions battery anodes: (**a**) potential profile in time in galvanostatic cyclations for cycles 1, 2, 5, and 10 at C/10; (**b**) capacitance calculated at different currents at C/10, C/5, C/2, C, and then back to C/10; (**c**) device life and coulombic efficiency.

Galvanostatic cyclations were performed at C/10 (assuming the material being comparable to graphite): the trend of potential in the first, second, fifth, and tenth cycles are shown in Figure 12a and performances at different currents are shown in Figure 12b. As can be noted, the first cycle is much longer than the others, likely due to the SEI formation occurring on the anode surface, and its discharging curve has a distinctly different shape in comparison to all the other cycles. More specifically, the decrease in potential at the beginning of the first cycle is less sharp than the decrease observed in the following cycles, in which the potential quickly fades around 1.5 V. SEI formation, however, still happens between 1.5 V and 1 V; therefore, we can conclude that there is a constant SEI formation during device operation. Considering their shape, the curves actually seem to indicate that different phenomena occur during the discharge, i.e., a first reaction below 1.5 V compatible with persistent SEI formation that decreases over cycling but never disappears, and a second reaction below 1 V compatible with lithiation of the small graphitic domain of graphene quantum dots. A possible explanation for this behavior could be a loss in cohesion of the active material and poor adhesion to the electrode. Despite having included a binder inside the electrode powder, GQDs apparently tend to detach from the rest of the material, thus exposing the underlying surface. This could lead to the irreversible formation of SEI even after the first cycle, which causes electrolyte consumption and a decrease in active surface, thus resulting in a continuous loss of capacity. The abovementioned considerations and experimental evidence have a direct effect on device life (Figure 12c). Even though the coulombic efficiency remains nearly constant at around 100%, the starting capacity, slightly below 120 mAhg^−1^, instantly drops at 100 mAhg^−1^. In the following cycles, the capacity fades linearly till around the 70th cycle, when the loss decreases: around the 100th cycle, the capacity tends to reach a plateau below 40 mAhg^−1^, with a retention of 38.6%. The obtained results are of course far from the capacity value of graphite, which is currently the golden standard for commercial applications. However, as already mentioned for supercapacitors, similar GQDs are rarely used as such: in the literature, remarkable results have so far only been reported for boron- or nitrogen-doped GQDs, or for GQDs combined with other active materials in hybrid systems [66]. Consequently, the present results should be considered as a starting point to evaluate the potential of the GQDs we propose. Their capability of intercalating lithium-ions should actually be considered an excellent starting point for their exploitation as electrode material, upon improvement of its adhesion to the current collector and the coupling with a second active component.

**Figure 13:**
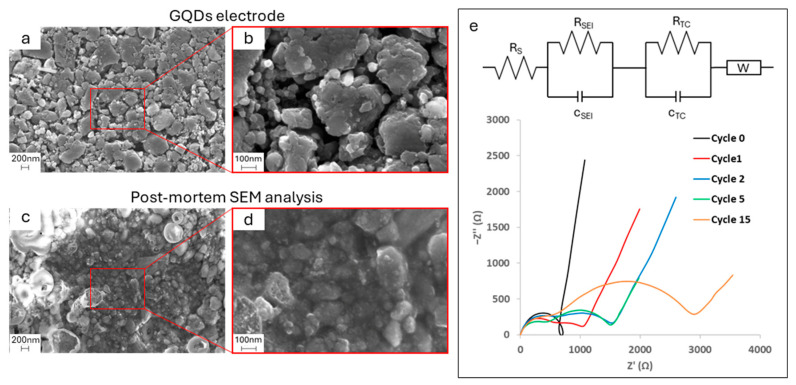
SEM micrographs of pristine GQDs at (**a**) 50k× and (**b**) 200k× magnification; SEM micrographs of post-mortem GQDs at (**c**) 50k and (**d**) 200k× magnification; (**e**) EIS analysis on GQD electrodes.

In order to prove this statement, both post-mortem SEM analysis and electronic impedance spectroscopy were performed, and the results are shown in Figure 13. SEM micrographs were taken after charging and show that, by moving from a pristine electrode (inserts a and b) to a post-cycled electrode after 50 cycles (inserts c and d), an inhomogeneous SEI is formed. There are regions where, due to gas formation during electrolyte degradation, SEI assumes a bubble-like morphology that covers the material underneath and other regions where the particles of GQDs visible in pristine samples are covered by a film. However, this non-uniform film seems to leave some areas uncovered, which, when facing the subsequent process of discharge, will serve as active sites for new SEI degradation.

The inhomogeneous formation of SEI and the continuous discovery of the underneath surface during cyclations is also confirmed by EIS spectroscopy, where EIS is collected on pristine device (cycle 0) and at four different moments, i.e., after the first, the second, the fifth, and the fifteenth cycles, as shown in Figure 13e. Before cyclations, the material shows a singular semicircle due to charge transfer phenomena, and right after the first cycle, when SEI is formed, a second semicircle appears, demonstrating the formation of a new layer above the electrode surface. This semicircle gets bigger after another cycle but it remains stable up to the fifth cycle, which means resistance is still kept low after a few cyclations; however, after this moment, due to the exposure of active material as shown by SEM post-mortem analysis, there is a further increase in resistance as the semicircle diameters become bigger after the fifteenth cycle. This increase in charge transfer phenomena resistance causes the capacitance to quickly fade.

Resistance values calculated using the equivalent circuit shown as a fitting model are shown in the Supplementary Materials (Table S1).

## 3. Materials and Methods

Rice husk was purchased by LD Carlson Co. (Kent, OH, USA) and was finely ground before use. All other chemicals were purchased from Sigma Aldrich (St. Louis, MO, USA) and were employed as is, without any further purification.

GQDs were synthesized starting from carbon aerogels (CAs) obtained by RH via an innovative synthesis already developed in our group [67]. Basically, RH underwent a bleaching in a 1.7% wt sodium chlorite and acetic acid aqueous solution (pH 3) to remove lignin and part of hemicellulose, followed by digestion in NaOH 1M at reflux temperature to remove silica and other inorganic impurities, together with the residual part of hemicellulose. The obtained cellulose pulp was dissolved into a 7% wt NaOH and 12% wt urea aqueous solution in an ice bath to obtain a final concentration of 7% wt of cellulose and aged at 50 °C until a stable cellulose gel was formed. Regeneration in water, freeze-drying, and carbonization in Ar atmosphere at 800 °C finally resulted in the formation of a carbon aerogel.

CA samples were ball-milled at 500 rpm for 1 h for 5 times and the final GQD powder was collected using pure water to rinse the ball milling jar.

Morphological characterization was performed using field emission scanning electron microscopy (FESEM) and atomic force microscopy (AFM). FESEM images were acquired by using a ZEISS Auriga platform (Oberkochen, Germany) equipped with energy-dispersive X-ray spectroscopy (EDX). FESEM analysis was performed both on freshly prepared materials and electrodes along with post-mortem samples after cyclations in lithium-ion devices. AFM analysis was performed in tapping mode air by using a BRUKER Dimension Icon (Bremen, Germany) and RTESP-300 BRUKER tips (300 kHz frequency, 40 N/m spring constant and nominal radius of 8 nm). Suitable samples were prepared by depositing a few drops of a diluted suspension (1:104 starting from a 0.4 g/L solution) of graphene quantum dots in water over a (100) crystalline silicon wafer and drying the wafer in air. The recorded images were postprocessed for background removal and leveling using the software Gwyddion version 2.62.

A F200 JEOL Multipurpose (Tokyo, Japan) transmission electron microscope (TEM) was used in high-resolution (HRTEM) mode to further investigate the morphology and crystalline structure with a higher accuracy. Analysis was conducted at 80 kV with an emission current of 134.7 mA. The camera used was a Gatan Rio16 model (Pleasanton, CA, USA). Suitable samples were prepared by depositing a few drops of a diluted suspension (1:106 starting from a 0.4 g/L solution) of graphene quantum dots in water over a dedicated TEM grid.

Dimensional distributions in SEM, AFM, and TEM were evaluated using ImageJ software, version 1.54j.

X-ray Diffraction (XRD) for structural analysis was performed using a Rigaku Miniflex diffractometer (Cedar Park, TX, USA) with Cu-Kα radiation (λ 1.54 Å) in 2θ range 8–40°, at 30 kV voltage and 15 mA of current. Raman spectroscopy was performed with a Renishaw (Wotton-under-Edge, UK) inVia Raman confocal Microscope using a green lamp (532.1 nm, output power 50 mW) and 100× lens, in the Raman shift range between 800 and 2200 cm^−1^. FTIR analysis was performed using a Bruker Lumos II micro-FTIR ATR equipped with Ge crystal with 4 cm^−1^ resolution and 2048 data points scan.

X-ray photoelectron spectroscopy (XPS) measurements were carried out using an Omicron (Uppsala, Sweden) NanoTechnology Multiprobe MXPS system equipped with a monochromatic Al Kα (hν = 1486.7 eV) X-ray source (Omicron XM-1000), operating the anode at 14 kV and 16 mA. The C 1s photoionization region was acquired using an analyzer pass energy of 20 eV. A take-off angle of 21° with respect to the sample surface normal was adopted. CA and GQD powders were spread onto double-sided conductive scotch tape attached to the XPS sample holder. The experimental spectra were theoretically reconstructed by fitting the secondary electron background to a Shirley function and the elastic peaks to pseudo-Voigt functions described by a common set of parameters: position, full width at half maximum (FWHM), and the Gaussian–Lorentzian ratio. The oxygen content was determined through the R_O/C_ ratio obtained after the curve fitting of the C 1s region, by means of the following equation:

(4) $$R_{O/C}=\frac{A_{C-OH}+1/2A_{C-O-C}+A_{C=O}+2A_{COOH}}{A_{C=C}+A_{C-OH}+A_{C-O-C}+A_{C=O}+A_{COOH}}$$

where the terms *A_x_* represent the OFG peak areas obtained by curve fitting.

Simultaneous thermogravimetric (TG-DTA) curves were registered using the TA Instruments Q650 system analyzer (Waters, Milford, MA, USA) thermal analysis equipment. The temperature was calibrated using the nickel Curie point as a reference, and the DTA baseline was calibrated using sapphire. Samples of about 8 mg in weight were placed in a high-purity alumina crucible and tested with a thermal profile consisting of a heating ramp from room temperature to 800 °C, at a heating rate of 10 °C min^−1^. Measurements were performed in technical air at a gas flow rate of 100 mL min^−1^. Exothermic peaks are conventionally up.

Electrochemical performances of graphene quantum dots were evaluated using a multichannel VMP potentiostat by Perkin Elmer Instruments (Waltham, MA, USA). Electrode powder was obtained upon mixing GQDs with PVDF and acetylene black in a proportion of 80:10:10 *w*/*w*. The electrodes were prepared upon suspending the powder in a slurry of N-Methyl-2-pyrrolidone (NMP) and drop-casting it either over platinum (for supercapacitor) or copper (for Li-ion batteries) current collectors. The chosen electrolytes were 6 M aqueous KOH for supercapacitors and LiPF_6_ in EC:DMC 1:1 for lithium-ion batteries. Two-electrode T-shaped cells were assembled in a symmetric configuration for supercapacitors, and in a half-cell configuration for Li-ion batteries. Galvanostatic cyclations and cyclic voltammetry tests for supercapacitors were performed in −1.0–0.2 V potential window, respectively, at different currents (0.1–1.0 Ag^−1^) and potential scan rates (1–50 mVs^−1^). In the case of Li-ion batteries, galvanostatic cyclations were performed in the range 0.04–3 V. Electrochemical impedance spectroscopy (EIS) was performed in the range of frequency from 1 MHz to 1 mHz with 10 mV of amplitude.

## 4. Conclusions

An efficient one-step way to produce graphene quantum dots from carbon aerogels was developed, and the final result was a fine powder of graphene quantum dots with an average diameter of around 20 nm as shown by TEM analysis. The GQD surface seemed functionalized due to the air atmosphere where the synthetic process occurred, while their structure in long and short range appeared to be made of short domains of graphitic nature generated by a crystallization process happening during synthesis. A preliminary study concerning the application of GQDs as both supercapacitor electrodes and lithium-ion anodes showed that the material has quickly fading performances probably due to poor contact with the rest of the electrode mixture and the current collector. However, graphene quantum dots proved to be able to both accumulate charges over the surface and intercalate ions, thus resulting in a promising 0D material worth deeper study in hybrid compounds.

## Figures and Tables

**Table 1:** Comparison of the material from the present work with the literature-reported electrodes made of 0D nano-dimensional carbon structures.

Materials	Capacitance [Fg^−1^]	Authors
GQDS	55	Present work
CD	19.00	Ref. [62]
CD bis-16	17.64	Ref. [62]
CD bis-64	32.08	Ref. [62]
CDs	30.5	Ref. [63]
CQDs	2.12	Ref. [64]

## Data Availability

No new data were created.

## Supplementary Materials

The following supporting information can be downloaded at: https://www.mdpi.com/article/10.3390/molecules29235666/s1, Figure S1: Gaussian fitting of the Raman spectrum of CAs; Figure S2: Gaussian fitting of the Raman spectrum of GQDs; Table S1: Evaluated resistance values obtained by fitting EIS spectra for lithium-ion batteries electrodes.
