# Supplementary material for: Graphene Quantum Dots from Agricultural Wastes: Green Synthesis and Advanced Applications for Energy Storage
Source: Molecules. 2024 Nov 29;29(23):5666. doi: 10.3390/molecules29235666 (PMC11643082; doi:10.3390/molecules29235666)
Supplement: Supplementary file 1 [file molecules-29-05666-s001.zip › molecules-3211597-supplementary.pdf]

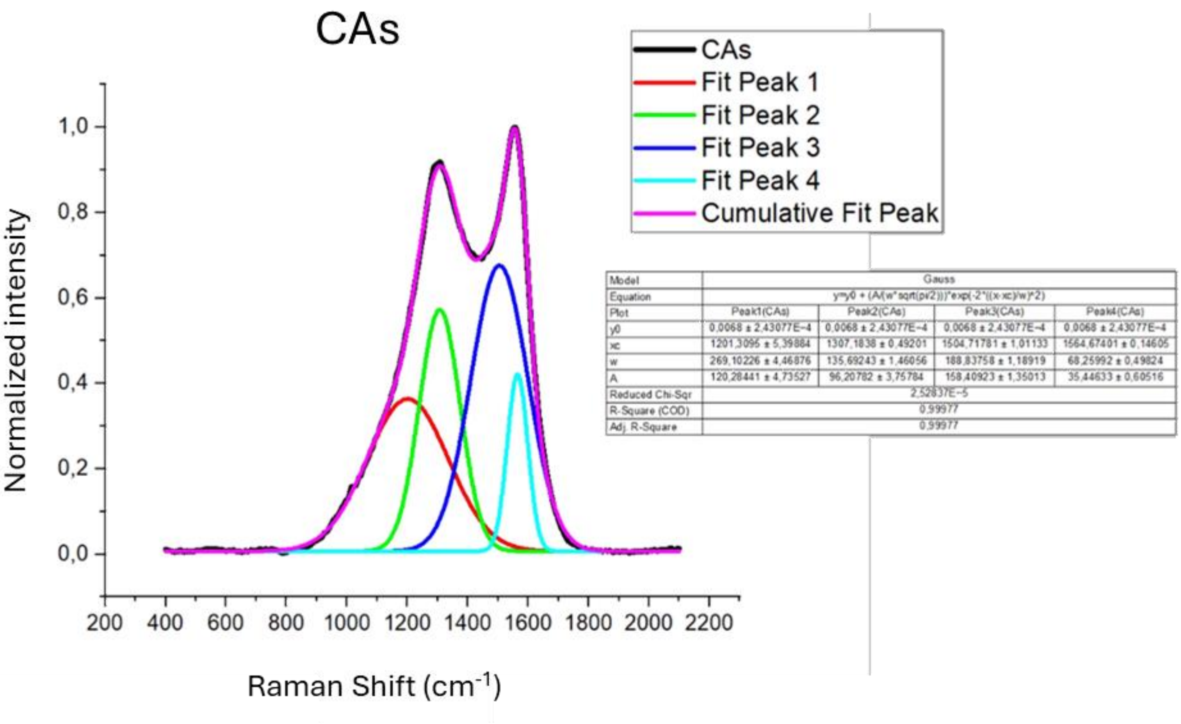

Model	Gauss			
Equation	y=y0 + (A/(w*sqrt(pi/2)))*exp(-2*((x-xc)/w)^2)			
Plot	Peak1(CAs)	Peak2(CAs)	Peak3(CAs)	Peak4(CAs)
y0	0,0068 ± 2,43077E−4	0,0068 ± 2,43077E−4	0,0068 ± 2,43077E−4	0,0068 ± 2,43077E−4
xc	1201,3095 ± 5,39884	1307,1838 ± 0,49201	1504,71781 ± 1,01133	1564,67401 ± 0,14605
w	269,10226 ± 4,46876	135,69243 ± 1,46056	188,83758 ± 1,18919	68,25992 ± 0,49824
A	120,28441 ± 4,73527	96,20782 ± 3,75784	158,40923 ± 1,35013	35,44633 ± 0,60516
Reduced Chi-Sqr	2,52837E−5			
R-Square (COD)	0,99977			
Adj. R-Square	0,99977			

Figure S1. Gaussian fitting of the Raman spectrum of CAs.

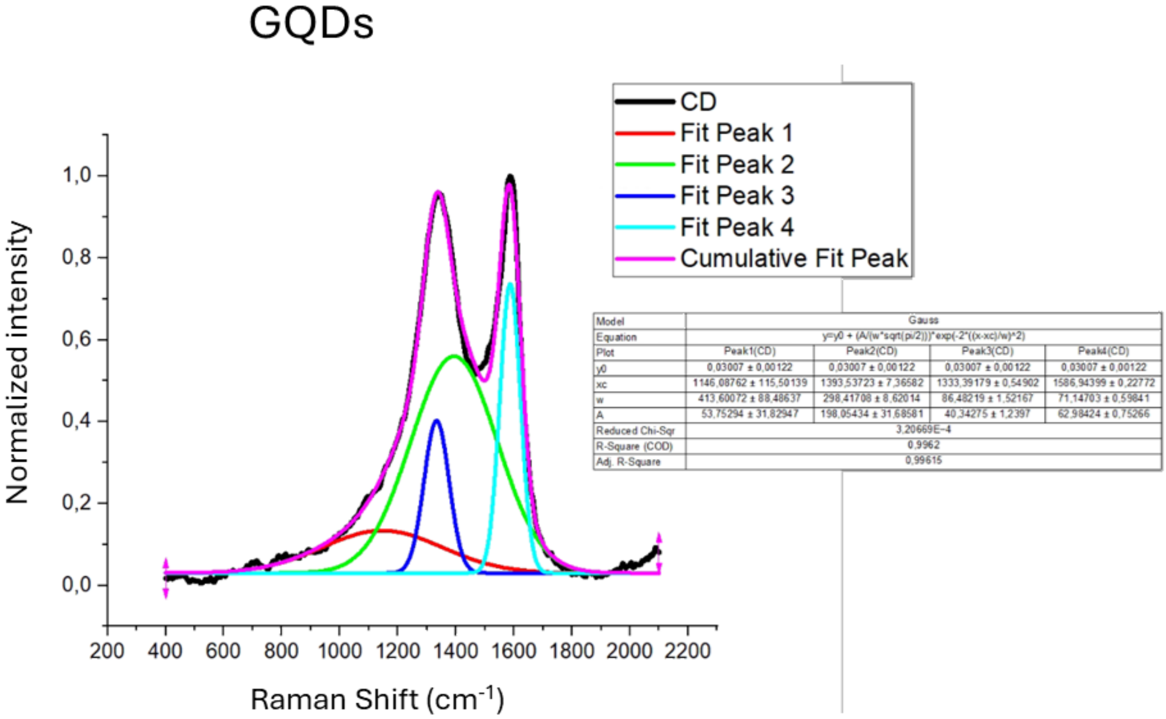

Model	Gauss			
Equation	y=y0 + (A/(w*sqrt(pi/2)))*exp(-2*((x-xc)/w)^2)			
Plot	Peak1(CD)	Peak2(CD)	Peak3(CD)	Peak4(CD)
y0	0,03007 ± 0,00122	0,03007 ± 0,00122	0,03007 ± 0,00122	0,03007 ± 0,00122
xc	1146,08762 ± 115,50139	1393,53723 ± 7,36582	1333,39179 ± 0,54902	1586,94399 ± 0,22772
w	413,60072 ± 88,48637	298,41708 ± 8,62014	86,48219 ± 1,52167	71,14703 ± 0,59841
A	53,75294 ± 31,82947	198,05434 ± 31,68581	40,34275 ± 1,2397	62,98424 ± 0,75266
Reduced Chi-Sqr	3,20669E−4			
R-Square (COD)	0,9962			
Adj. R-Square	0,99615			

Figure S2. Gaussian fitting of the Raman spectrum of GQDs.

Table S1. Evaluated resistance values obtained by fitting EIS spectra for lithium ion batteries electrodes.

cycle	RS (ohm)	CSEI (F)	RSEI (ohm)	CCT (F)	RCT (ohm)	S1 (ohm s^1/2)
0	2.28			1.36E-06	802.90	105.00
1	2.23	1.51E-06	499.20	1.39E-05	426.70	104.70
2	2.19	1.20E-06	485.40	2.28E-05	743.70	127.80
5	2.26	6.05E-05	357.90	1.16E-06	863.00	65.06
15	2.77	1.19E-06	404.20	49.53	1848.00	97.76
